# Upregulation of NPL4 promotes bladder cancer cell proliferation by inhibiting DXO destabilization of cyclin D1 mRNA

**DOI:** 10.1186/s12935-019-0874-2

**Published:** 2019-05-30

**Authors:** Bao-Sai Lu, Yue-Wei Yin, Yan-Ping Zhang, Ping-Ying Guo, Wei Li, Kai-Long Liu

**Affiliations:** Department of Urology, The Second Hospital of Hebei Medical University, No. 215, Heping West Road, Shijiazhuang, 050000 Hebei People’s Republic of China

**Keywords:** NPL4, DXO, mRNA stabilization, Proliferation, Bladder cancer

## Abstract

**Background:**

NPL4 is an important cofactor of the valosin-containing protein (VCP)–NPL4–UFD1 complex. The VCP–NPL4–UFD1 has been considered as a ubiquitin proteasome system (UPS) regulator and response to protein degradation. While NPL4 plays important roles in various diseases, little is known about its functions in bladder cancer (BC).

**Methods:**

MTT assays and colony forming test were performed to evaluate cell proliferation ability and Western blotting was used to detect protein expression. Cyclin D1 mRNA expression was detected using qRT-PCR, and coimmunoprecipitation (CoIP) was used to detect protein–protein interactions.

**Results:**

NPL4 was upregulated in BC tissue and correlated with poor prognosis. Upregulation of NPL4 promoted cell proliferation while suppression of NPL4 reduced BC cell proliferation. Upregulation of NPL4 led to overexpression of cyclin D1 by enhancing its mRNA stability. Moreover, NPL4 was found to bind directly to DXO and induce its degradation. DXO was downregulated in BC tissue and regulated BC cell proliferation by destabilizing cyclin D1 mRNA. DXO-mediated NPL4 regulated BC cell proliferation by stabilizing cyclin D1 expression.

**Conclusions:**

The NPL4/DXO/cyclin D1 axis exert crucial role in BC cell growth and is associated with prognosis and may represent a potential therapeutic target for BC.

## Background

Bladder cancer (BC) is the fourth commonly diagnosed cancer in men and the 11th most frequently diagnosed cancer in women [1]. Last year, 79,030 new BC patients and 16,870 cancer-related deaths were reported in the United States [2]. The most frequent histological subtype of BC is urothelial carcinoma which classified into two major types: non-muscle invasive bladder cancer (NMIBC) and muscle-invasive bladder cancer (MIBC) [3]. Approximately 70% of all BC cases are NMIBC [4] and around 50% of NMIBC patients exhibit recurrence after transurethral resection [5]. Many patients progress to muscle-invasive disease due to multiple recurrences [6]. Therefore, understanding the molecular mechanism of bladder tumorigenesis and discovering new treatment strategies are urgently required to improve diagnosis and treatment of BC [7, 8].

Characterization of the molecular landscape of poorly differentiated BC has enabled evaluation of the clonal evolution of BC progression [9]. Deregulation of genes can result from epigenetic silencing [10], transcriptional repression [11], post-transcriptional regulation [12] and cellular protein degradation [13] and so on. The ubiquitin–proteasome pathway is the most critical way involved in the regulation of several proteins with fundamental functions relevant to tumorigenesis, such as cell-cycle progression, apoptosis and gene transcription [14]. Nuclear protein localization protein 4 homolog (NPL4), an important partner of ubiquitin fusion degradation-1 (UFD1), was first identified in a selection for mutants defective in nuclear import [15]. UFD1–NPL4 heterodimer functions either alone or bound to the ubiquitous, p97 or valosin-containing protein (VCP) in higher eukaryotes. The VCP–UFD1–NPL4 complex plays an essential role in ubiquitin–proteasome-dependent degradation by transporting the polyubiquitin-tagged proteins to the 26S proteasome for processive degradation [16]. VCP–UFD1–NPL4 was shown to cause a severe phenotype, including cell cycle regulation, DNA damage response and genomic stability [17]. But the role of NPL4 in BC remains unclear.

In eukaryotic, stability of mRNA mainly depends on the 5′ RNA cap structure (^m7^GpppRNA). Meanwhile, the 5′ RNA cap structure exerts key roles in polyadenylation, splicing, mRNA export and translation [18–20]. Decapping enzyme is associated with mRNA decay, turnover and quality control as the 5′ monophosphorylated RNA is rapidly degraded [21]. Decapping and exoribonuclease protein (DXO), known as Rai1/Ydr370c and Dom3Z in fungal species and mammals, is a component of the mRNA 5′-end capping quality control mechanism and displays several biochemical functions such as decapping, pyrophosphohydrolase, deNADding, and 5′–3′ exoribonuclease activities [22]. Bioinformatic analyses and gene expression assays suggest that human Rai1 family correlate with gene transcription directly or indirectly. However, the downstream genes of DXO and its biological roles in BC are still unclear.

In the present study we analyzed NPL4 mRNA expression from the TCGA database as well as in clinical samples and found that NPL4 expression was significantly increased in BC tissues. We demonstrated that upregulation NPL4 binds directly to DXO and induces its degradation, whereas DXO regulates bladder cancer cell proliferation via destabilization of cyclin D1 mRNA. Our findings provide evidence that the NPL4/DXO/cyclin D1 regulatory axis may be responsible for BC progression.

## Results

### NPL4 is upregulated in BC tissues and is associated with poor prognosis

**Fig. 1:**
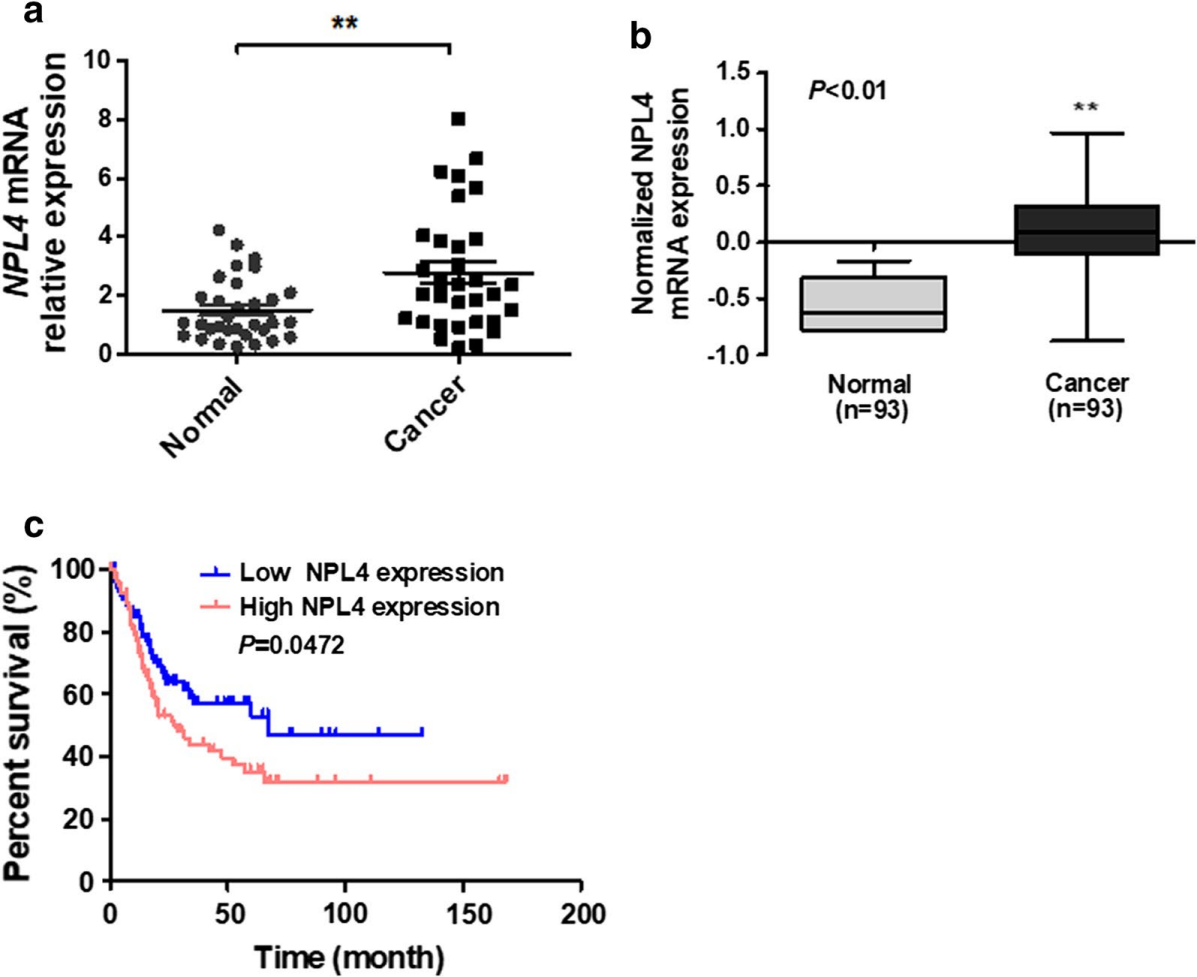
NPL4 is upregulated in BC tissue and is associated with poor prognosis. **a** qRT-PCR analysis detected NPL4 mRNA expression in BC (n = 35) and normal bladder (n = 35) tissues. **P < 0.01 vs. normal bladder tissue. **b** Expression of NPL4 mRNA in BC tissues and normal tissues from the GSE13507 database. **c** Kaplan–Meier analysis was used to analyze the overall survival of BC patients with low or high NPL4 expression

To identify whether NPL4 level were changed in BC tissue, qRT-PCR analysis was used to validate NPL4 mRNA expression in BC and normal bladder tissues. The result revealed NPL4 mRNA level in BC tissues was higher compared with that of normal bladders (Fig. 1a). Additionally, the results was confirmed by the microarray data from the TCGA database, and found that NPL4 mRNA expression was increased in BC tissue (Fig. 1b). Using the TCGA database, data from Oncolnc (http://www.oncolnc.org/) human clinical sample surveys also showed that patients with higher NPL4 mRNA expression had a significantly poor overall survival (*P *< 0.0001, Fig. 1c). In order to explore the clinical significance of NPL4 in bladder cancer, we analyzed the NPL4 expression pattern in 35 bladder cancer specimens by real-time PCR. The correlation analysis of NPL4 expression revealed NPL4 expression significantly associated with tumor size (*P *= 0.035) and tumor multiplicity (*P *= 0.041) (Table 1). However, there was no significant correlation between NPL4 expression and other clinicopathologic factors, such as age, sex, tumor grade, T classification, or metastasis. These findings indicate that upregulation of NPL4 play a role in promoting BC progression.

**Table 1:** Correlation between NPL4 mRNA expression and clinicopathological characteristics

Characteristics	Number of patients (%)	NPL4 expression		P value^a^
		Low (%)	High (%)	
Total no. of patients	35	18	17	
Age (years)				
≤ 63^b^	17	9 (52.94)	8 (47.06)	1.000
> 63	18	9 (50.00)	9 (50.00)	
Gender				
Male	22	12 (54.55)	10 (45.45)	0.733
Female	13	6 (46.15)	7 (53.85)	
Tumor size (cm)				
≤ 3.0^c^	22	14 (63.64)	8 (36.36)	*0.035*
> 3.0	13	3 (23.08)	10 (76.92)	
Tumor grade				
Low	21	10 (45.45)	11 (54.55)	0.733
High	14	8 (57.14)	6 (42.86)	
T classification				
Ta, T1	23	13 (56.52)	10 (43.48)	0.489
T2–T4	12	5 (41.67)	7 (58.33)	
pN status				
pN−	27	13 (48.15)	14 (51.85)	0.691
pN+	8	5 (62.50)	3 (37.50)	
Tumor multiplicity				
Unifocal	15	11 (41.67)	4 (41.67)	*0.041*
Multifocal	20	7 (41.67)	13 (41.67)	

Significant associations are shown in italic face in the P value column (P value < 0.05)

^a^Chi square test

^b^Median age

^c^Median size

### NPL4 plays an essential role in BC cell proliferation

**Fig. 2:**
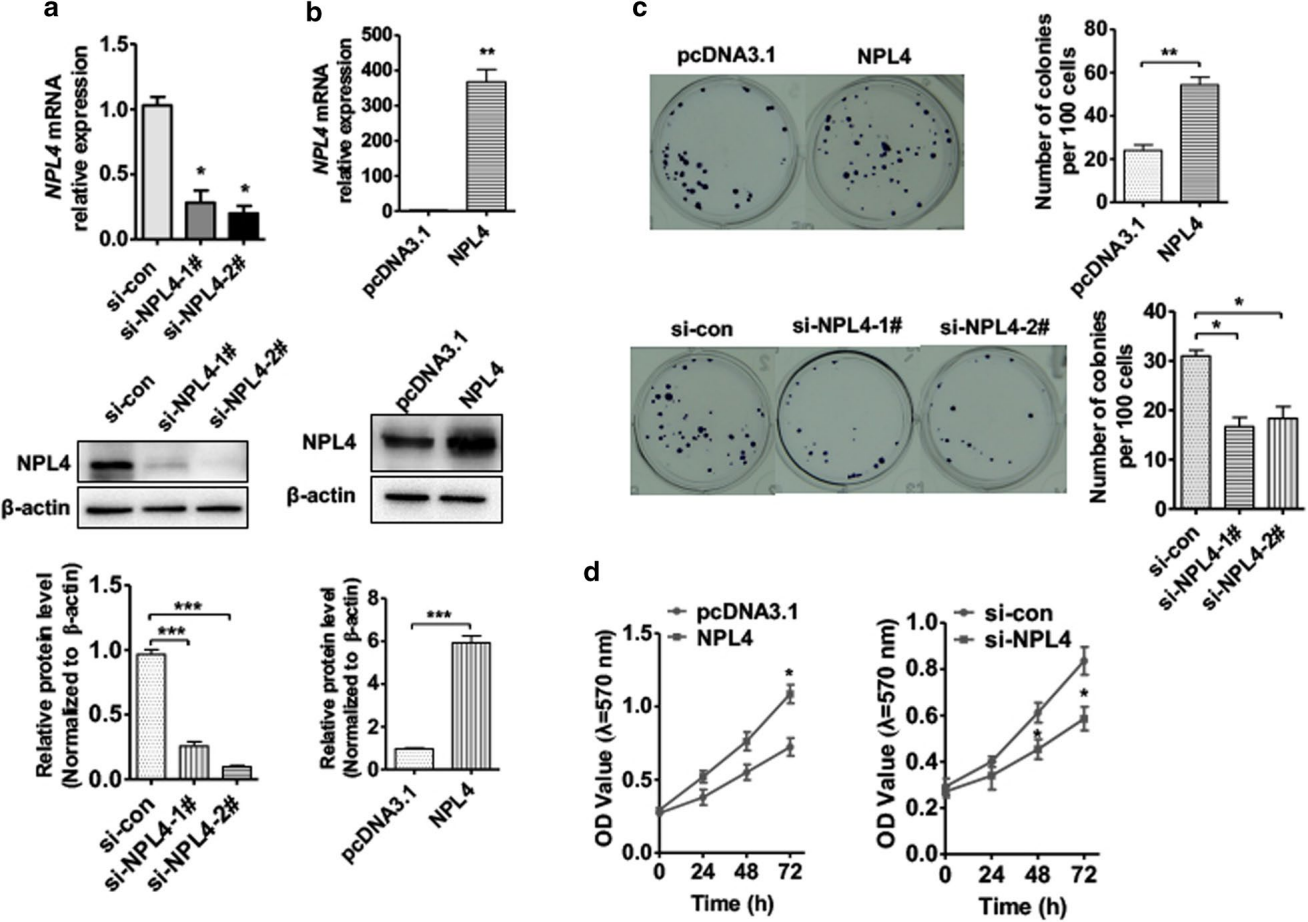
NPL4 plays an essential role in BC cell proliferation. **a** T24 cells were transfected with si-NPL41, si-NPL4-2, or negative control (si-NC). Western blot analysis and qRT-PCR were used to examine NPL4 expression. Bottom panel shows densitometric analysis of three independent experiments. ***P < 0.001 vs. si-NC. **b** T24 cells were transfected with NPL4 overexpression vector pcDNA3.1–NPL4 or empty vector. Western blot analysis and qRT-PCR were used to examine NPL4 expression. Bottom panel shows densitometric analysis. **P < 0.05, ***P < 0.01 vs. empty vector. **c**, **d** T24 cells were prepared as in **a** and **b**, and MTT and colony formation assays were used to detect the cell viability. Right panel shows colony number analysis. ***P < 0.01 vs. corresponding control

To explore the biological functions of NPL4 in BC, T24 cells were transfected with the NPL4 overexpression vector, pcDNA3.1–NPL4, or small interfering RNAs (siRNAs) against NPL4, using empty vector or control si-RNA as negative controls, respectively. si-NPL4 transfection led to a dramatic downregulation of NPL4 mRNA and protein levels compared with si-control RNA in T24 cells (Fig. 2a, b). On the contrary, transfection with pcDNA3.1–NPL4 in T24 cells significantly upregulated NPL4 mRNA and protein levels. Next, colony formation assays and MTT assays were used to investigate the effect of NPL4 on BC cell proliferation. Colony formation assays revealed lower proliferation of T24 cells transfected with si-NPL4 compared with cells transfected with negative control (Fig. 2c). In contrast, overexpression of NPL4 significantly increased proliferation of T24 cells compared with the empty vector. As expected, the MTT assays further confirmed that knockdown of NPL4 expression in T24 cells decreased proliferation ability compared with control si-RNA. On the contrary, T24 cells overexpressing NPL4 showed an increased proliferation ability compared with control cells (Fig. 2d). These findings revealed that overexpression of NLP4 may be correlated with BC cell proliferation.

### NPL4 promotes BC cell proliferation by regulating cyclin D1 mRNA stability

**Fig. 3:**
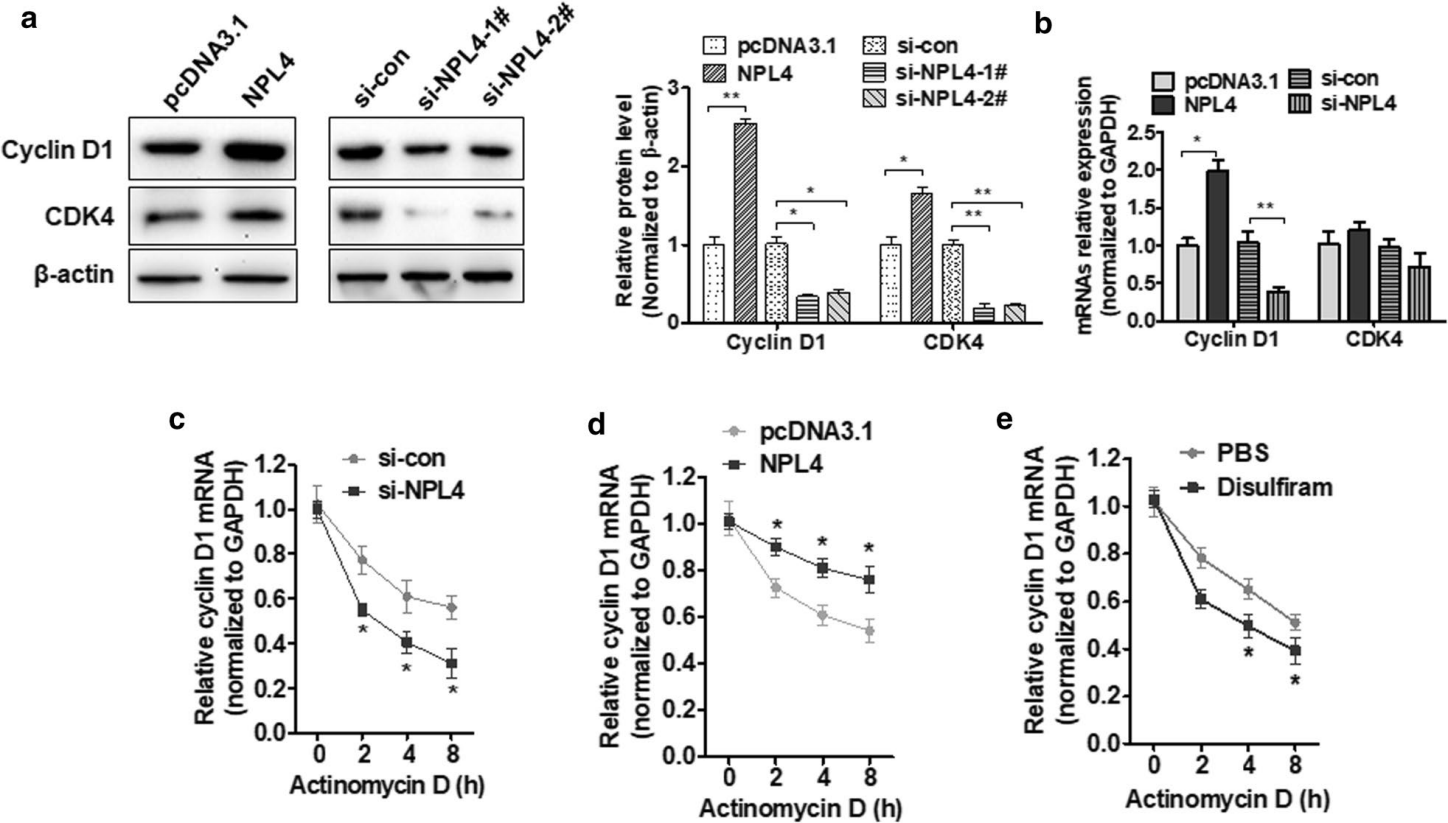
NPL4 promotes proliferation of BC cell by regulating cyclin D1 mRNA stability. **a**, **b** T24 cells were transfected with NPL4 overexpression vector or si-NPL4. Western blot analysis and qRT-PCR analysis were performed to exam cyclin D1 and CDK4 protein and mRNA levels, respectively. **c** T24 cells were transfected with si-NPL4 or negative control (si-NC) then exposed to ActD for 2, 4, 6, and 8 h. Cyclin D1 mRNA levels were detected by qRT-PCR. **d** T24 cells were transfected with pcDNA3.1–NPL4 or empty vector then exposed to ActD for 2, 4, 6, and 8 h. qRT-PCR analysis was used to exam Cyclin D1 mRNA levels. *P < 0.05, **P < 0.01 vs. control group. **e** T24 cells were treated with the NPL4 inhibitor, disulfiram, and then exposed to actinomycin D with for different lengths of time. Cyclin D1 mRNA levels were detected by qRT-PCR

A previous study reported that NPL4 was associated with cell cycle by promoting CDC25A protein degradation [23]. We next investigated the mutual relationship between NPL4 and cell cycle-associated gene expression in BC cells. T24 cells were transfected with pcDNA3.1–NPL4 or si-NPL4. Cyclin D1 and CDK4 expression were detected by qRT-PCR and Western blotting. Overexpression of NPL4 significantly increased protein levels of cyclin D1 and CDK4, as well as cyclin D1 mRNA, but not CDK4 mRNA. NPL4 depletion using si-RNA markedly reduced cyclin D1 and CDK4 expression (Fig. 3a, b). Since NPL4 plays an important role in protein degradation, we investigated how NPL4 regulates mRNA expression. To directly investigate the role played by mRNA stability, we measured cyclin D1 mRNA expression changes over time using qRT-PCR after blocking transcription with actinomycin D (ActD). Knockdown of NPL4 in T24 cells abolished the effects of ActD on cyclin D1 mRNA stability, whereas NPL4 overexpression enhanced the effects of ActD (Fig. 3c, d). Similarly, suppression of NPL4 by disulfiram, an inhibitor of NPL4, also reduced mRNA expression compared with the control (PBS) group (Fig. 3e). These finding suggest that NPL4 positively regulates cyclin D1 expression by stabilizing its mRNA.

### NPL4 interacts with DXO and suppresses DXO protein expression

**Fig. 4:**
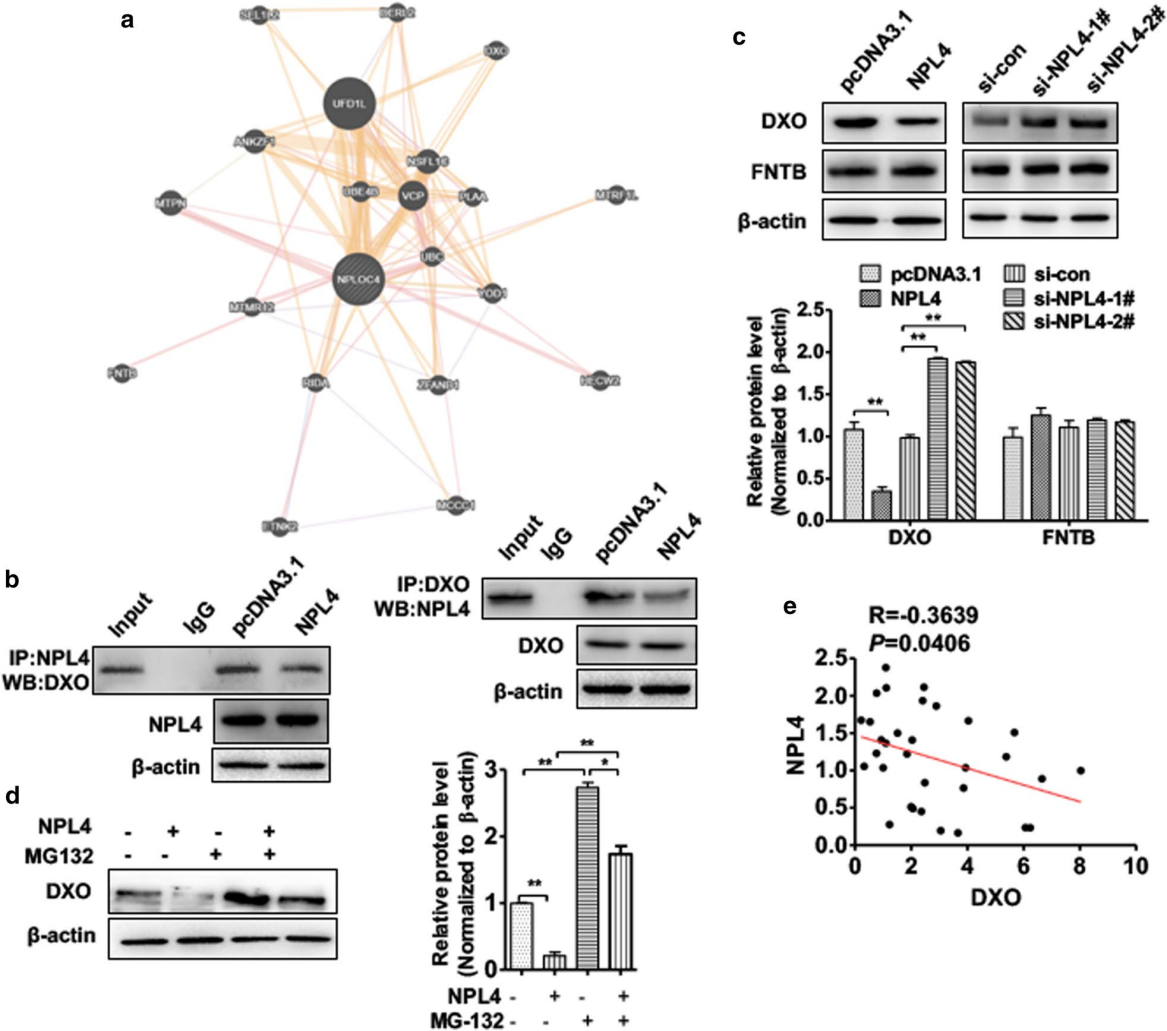
NPL4 interacts with DXO and negatively regulates DXO protein expression. **a** GeneMANIA used to analyzed network of proteins according to the databases. All the nodes were connected and related to NPL4. **b** T24 cells were transfected with si-NPL4 (or si-con) and pcDNA3.1-NPL4 (or empty vector). Western blot analysis was used to detect DXO protein levels. **c** T24 cells were transfected with NPL4 overexpression pcDNA–3.1NPL4 or empty vector. CoIP analysis was used to detect the interaction between DXO and NPL4. **d** T24 cell were treated with MG132 or DMSO for 6 h and then transfected with NPL4 overexpression pCDNA-3.1NPL4 or empty vector. DXO protein level was detected by western blot analysis. **e** The relationships between NPL4 and DXO was analyzed by Pearson correlation.(R = − 0.3639, *P *= 0.0406)

Our findings prompted us to investigate how NPL4 regulates mRNA stability. A previous study showed that VCP–Ufd1–NPL4 helps process ubiquitin-labeled proteins for recycling or degradation by proteasomes. As a ubiquitin-associated gene, NPL4 could regulate protein expression but not mRNA levels. We speculated that NPL4 may regulate a gene related to mRNA stabilization at the ubiquitination level that directly regulates cyclin D1 mRNA stability. First, we used GeneMANIA to construct interaction networks to analyze NPL4 associated with its interaction proteins and revealed a number of proteins directly or indirectly related to NPL4 (Fig. 4a). We focused on DXO, a decapping enzyme of mRNA. The GeneMANIA network showed that DXO formed a direct network with NPL4. To further confirm the relationship between DXO and NPL4, we performed CoIP after overexpressing NPL4 in T24 cells. CoIP of endogenous proteins confirmed interactions between NPL4 and DXO, but not FNTB. However, NPL4 overexpression decreased the interaction between NPL4 and DXO (Fig. 4b). To investigate whether NPL4 regulated DXO expression, we next performed a gain-and-loss of function experiment. Depletion of NPL4 expression in T24 cells significantly increased, whereas NPL4 overexpression decreased protein levels of DXO, but not FNTB (Fig. 4c). To further confirm NPL4 negatively regulated DXO protein level by promoting its degradation, we performed a rescued experiment. As shown in Fig. 4d, T24 cell treated with proteasome inhibitor MG132 led to a significant increase of DXO protein level. While overexpression of NPL4 in T24 cells then absence of MG132 could increase DXO protein level reduced by NPL4, indicating that NPL4 could down-regulate DXO by promoting the ubiquitin proteasome pathway. Moreover, increased levels of NPL4 correlated with a decrease in DXO expression in human BC tissue (Fig. 4e). These results indicated that DXO interacts with and is negatively regulated by NPL4.

### Knockdown of DXO promotes BC cell proliferation by stabilizing cyclin D1 mRNA

**Fig. 5:**
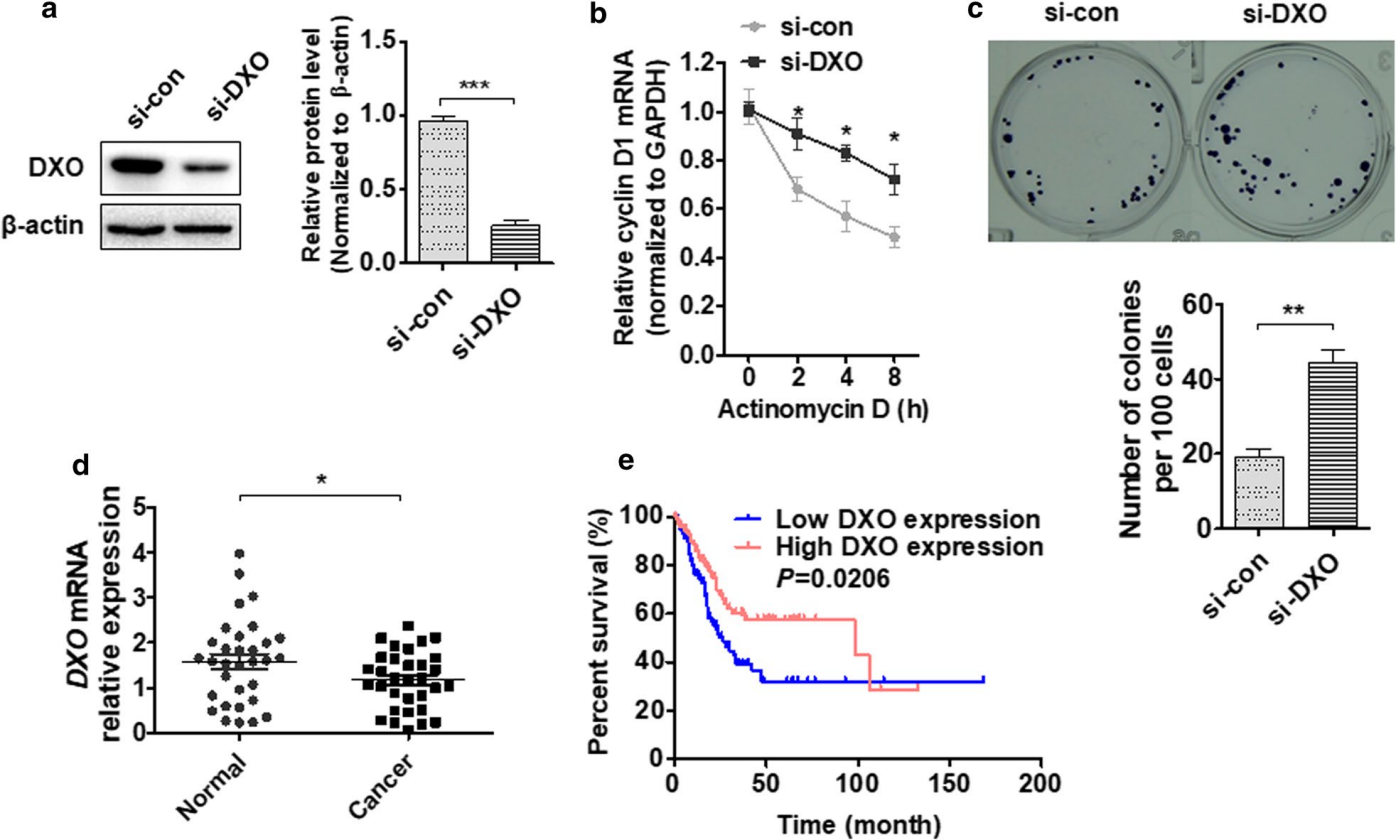
Knockdown of DXO promotes BC cell proliferation by stabilizing cyclin D1 mRNA. **a** T24 cells were transfected with si-DXO or negative control. Western blot analysis was performed to detect DXO protein level. Right panel shows densitometric analysis. ***P < 0.001 vs. empty vector. **b** T24 cells were transfected with si-DXO or si-con and then exposed to actinomycin D for 0, 2, 4, and 8 h. Cyclin D1 mRNA expression was detected using qRT-PCR. *P < 0.05 vs. si-con. **c** T24 cells were prepared as in **a**, colony formation assays were performed to measure proliferation ability. **d** qRT-PCR analysis detected DXO expression in BC and normal bladder tissues. *P < 0.01 vs. normal bladder tissue. **e** The overall survival of the BC patients was analyzed by Kaplan–Meier Upregulation of DXO was correlated with more a favorable overall survival (*P* = 0.0206)

To clarify the intrinsic relationship between DXO and cell proliferation, we knocked down DXO in T24 cells using si-RNA. We revealed that si-DXO transfection significantly reduced DXO protein levels compared control si-RNA (Fig. 5a). To demonstrate the involvement of DXO in cyclin D1 mRNA expression, T24 cells were transfected with si-DXO, followed by treatment with ActD. Knockdown of DXO increased cyclin D1 mRNA expression (Fig. 5b). Colony formation assays showed that DXO knockdown increased proliferation of T24 cells compared with the control group (Fig. 5c). In addition, we examined DXO mRNA expression in clinical tissue and found that DXO mRNA levels were significantly downregulated in BC compared with normal tissue (Fig. 5d). Further correlation analyses demonstrated that upregulated DXO levels were correlated with a reduced overall survival in patients with BC (P = 0.0026; Fig. 5e). These results further suggest that knockdown of DXO promotes bladder cell growth by stabilizing cyclin D1 mRNA.

### DXO-mediated NPL4 regulates BC cell proliferation

**Fig. 6:**
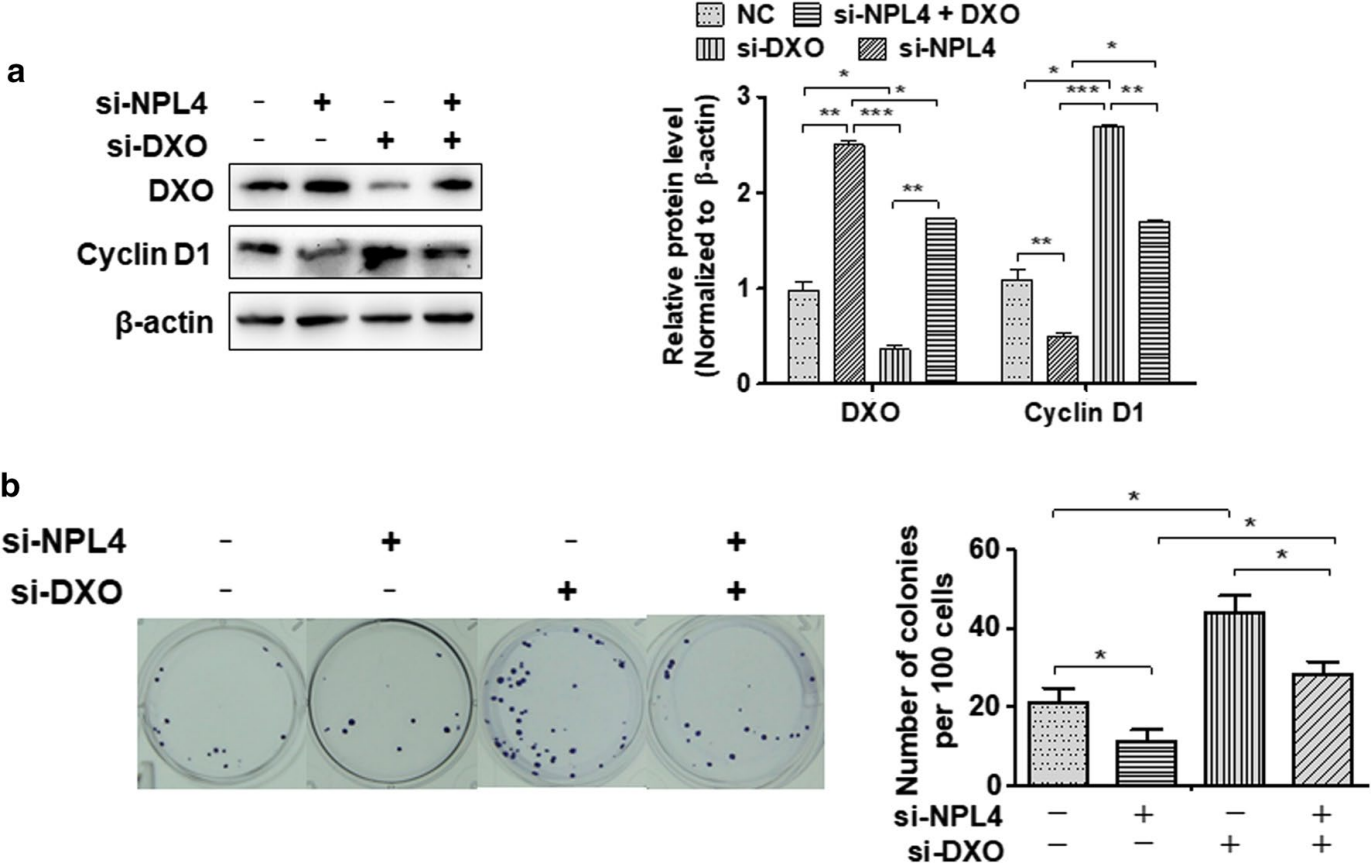
DXO-mediated NPL4 suppresses T24 cell proliferation by regulating cyclin D1 expression. **a** CoIP detected the interaction between DXO and NPL4 by using CDC48 antibody in NPL4-transfected T24 cell after treatment with or without MG-132. **b** qRT-PCR detected the DXO mRNA expression in T24 cells treatment as above. **c** T24 cells were transfected with siRNAs. DXO and cyclin D1 protein levels were detected by western blotting. Right panel shows densitometric analysis. *P < 0.05, ***P < 0.001 vs. corresponding controls. **d** T24 cells were prepared as above, and colony formation assays were used to exam cell viability. Up panel shows colony number analysis. *P < 0.05 vs. corresponding control

**Fig. 7:**
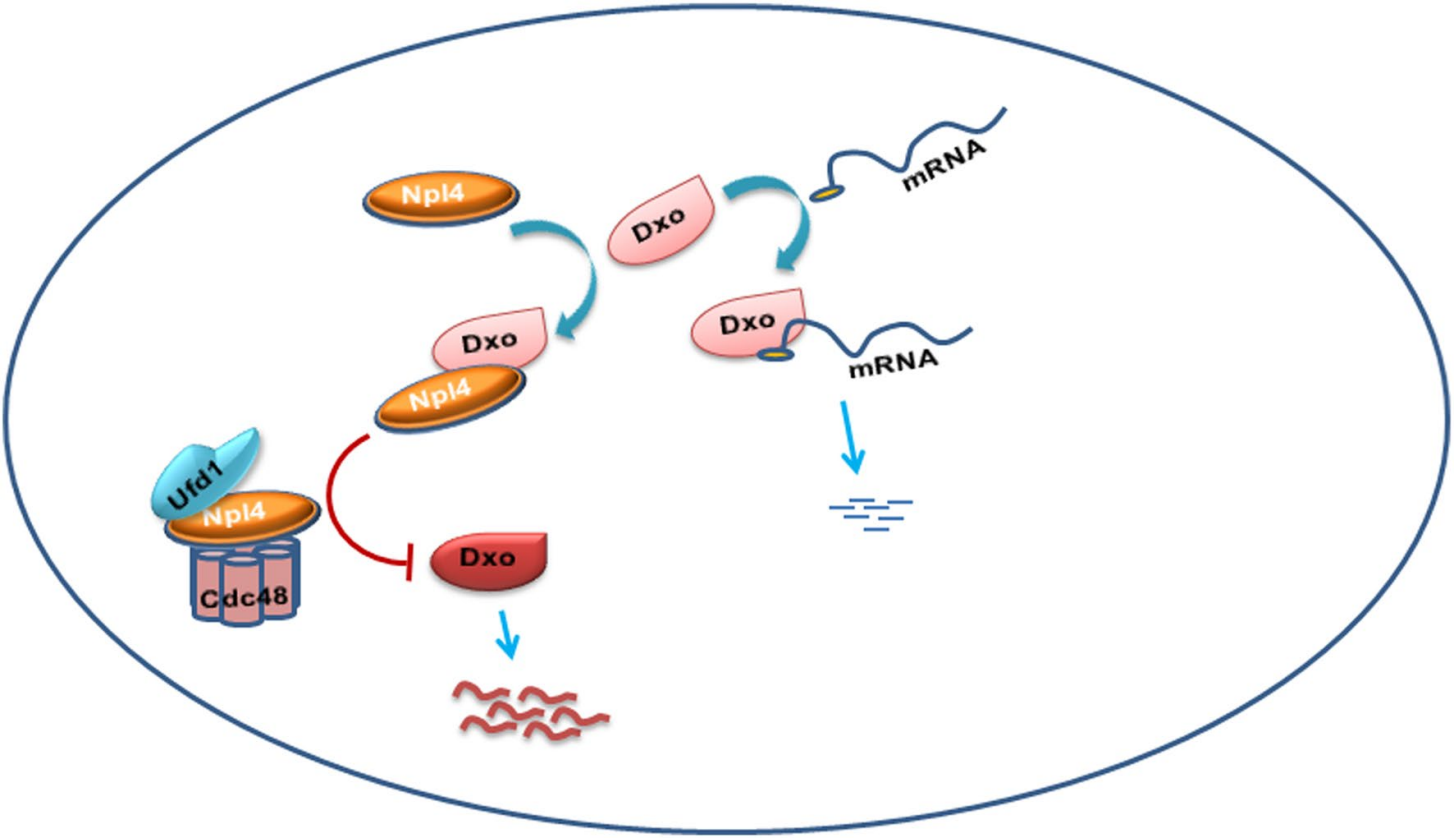
Proposed model for NPL4/DXO/Cyclin D1 axis regulation of BC proliferation. Upregulated NPL4 binds directly to DXO and induces its degradation, whereas DXO regulates BC cells proliferation by destabilizing cyclin D1 mRNA

To elucidate whether DXO mediates NPL4 regulation of BC proliferation, T24 cells were transfected with si-NPL4, si-DXO, or cotransfected both together. Western blot showed that cotransfection of si-NPL4 and si-DXO reversed the cyclin D1 decrease by knockdown of si-NPL4 alone (Fig. 6a, lanes 2 and 4). Colony formation assays further confirmed that simultaneous knockdown of NPL4 and DXO significantly increased proliferation activity in T24 cells compared with knockdown of NPL4 alone (Fig. 6b, lanes 2 and 4). These findings indicate that NPL4 induced cell proliferation by regulating the DXO/cyclin D1 axis in BC cells. Proposed model underlying the role of NPL4/DXO/cyclin D1 in BC cell is shown in Fig. 7.

## Discussion

The present study revealed that: (1) NPL4 is up-regulated in BC tissue and is correlated with poor prognosis, (2) overexpression of NPL4 promotes BC cell proliferation, while depletion of NPL4 reduces BC cell proliferation, (3) upregulation of NPL4 leads to elevation of cyclin D1 by enhancing its mRNA stability, (4) NPL4 binds directly to DXO and induces its degradation, and (5) the NPL4/DXO/cyclin D1 axis exerts an critical role in BC cell proliferation and associated with prognosis.

The ubiquitin proteasome system (UPS) is the primary protein processing system of the cell. Target proteins are initially recognized by upstream components and tagged with polyubiquitin chains. Then these polyubiquitinated proteins are send to 26S proteasome for degradation [24]. Many proteins that are regulated by ubiquitylation control cellular processes, such as apoptosis, cell-cycle progression and gene transcription that are relevant to tumorigenesis [25]. Deregulation of ubiquitin pathways results in the development of human diseases, including many types of tumors [26]. Previous studies focused extensively on ubiquitin ligases enzymes and deubiquitylating enzymes that modulate oncogenic signaling pathways [27]. Recent studies have focused on the VCP–UFD1–NPL4 complex that functions centrally in the ubiquitin–proteasome pathway. NPL4 is a 67-kDa protein that forms a stable heterodimer with UFD1, which, in turn, binds the ubiquitous p97/VCP ATPase [27]. Subsequently, the VCP–UFD1–NPL4 complex binds to ubiquitin-tagged proteins, distinguishing them from non-ubiquitinated proteins, and sends them to the proteasome for degradation [28]. In addition, the VCP–UFD1–NPL4 complex was also thought to release the ubiquitin chain from ubiquitylated proteins for either degradation or recycling [29, 30]. Substrates of VCP–UFD1–NPL4-mediated UPS include transcription factors [31] robust G_2_/M checkpoint [32], cell cycle regulators, cell death [33], mitochondrial targets for the clearance of damaged mitochondria [34] and aggregate prone proteins [35]. Despite the fact that in many instances UFD1 and NPL4 work together in UPS-dependent protein degradation, UFD1–NPL4 promotes the retro-translocation of emerging endoplasmic reticulum (ER) proteins for degradation in a process known as ER-associated degradation [36]. Surprisingly, VCP–UFD1–NPL4 pathway is a promising therapeutic target in oncology. Disulfiram, an effective alcohol-aversion drug, demonstrated anti-tumor effects by targeting VCP–UFD1–NPL4 pathway involved in multiple regulatory and stress-response cellular pathways [37]. In the present study, we confirmed NPL4 was up-regulated in BC tissues and associated with poor prognosis. Depletion of NPL4 in T24 cells reduced cell growth. Our study demonstrated that NPL4 functions as an oncogene by regulation cyclin-D1 expression in BC. Importantly, disulfiram (an NPL4 inhibitor), downregulated cyclin-D1 expression. Therefore, NPL4 might be a potential treatment for BC. However, the activity of VCP and expression of its co-factor, UFD1, remain unknown. It is unclear whether NPL4 may become a mature biological marker. Furthermore, the anti-BC effects of disulfiram as well as its molecular mechanism require further investigations.

Regulation of gene expression is a coordinated and multi-layered process involving many trans-acting factors. During the last decade, accumulated evidence showed that mRNA stability played a central role in cell differentiation, proliferation and adaptation [38]. In eukaryotes, mRNAs are first synthesized in the nucleus as pre-mRNAs that are subject to 5′-end capping, splicing, 3′-end cleavage, and polyadenylation [39]. RNA-binding proteins and non-coding RNAs interact with mRNAs in a combinatorial manner and coordinate post-transcriptional regulation to achieve appropriate spatio-temporal expression of the encoded proteins [39–42]. However, the lifespan of mRNA lives depends on how efficiently the mRNA degradation machinery is recruited to that pre-mRNA [39]. A central premise in eukaryotic mRNA metabolism is the addition of an m^7^G cap at the 5′ end of the mRNA that promotes its translation and stability [43]. Removal of the 5′ end cap of pre-mRNA is a efficient controlling mRNA stability and meaningful for gene transcription. Because of the decapping enzymes which has the enzyme activity to remove the cap structure from the 5′ end of mRNA transcripts [44, 45], decapping enzymes has been reported closely related to the development in multiple diseases. For example, decapping enzyme Nudt3, a member of nudix hydrolase superfamily, promoting MCF7 cell migration and proliferation by modulating β6 and lipocalin-2 mRNA stability [46]; Dcp2, the first discovered decapping enzyme, enhanced tumor cell growth by affected RAS and MYC mRNA stability [47].

DXO is a mammalian homolog of Rai1 and Dxo1 that possesses pyrophosphohydrolase, decapping, and 5′–3′ exoribonuclease activities [22]. These findings implicate DXO as a crucial arbiter of the co-transcriptional capping process in mammalian cells. The large increase in partially capped pre-mRNA population upon DXO knockdown in vivo suggests that the enzyme identifies, decaps, and degrades a significant number of incompletely capped Pol II transcripts [21]. Therefore, DXO functions as an important quality control mechanism of pre-mRNA capping [22]. However, the significance and specific mechanisms of DXO have not been extensively studied. In the present study, we first confirmed that DXO expression is downregulation in human BC and corresponding normal bladder tissues. According to the TCGA database, lower expression of DXO was significantly associated with shorter survival in BC patients. Using a series of in vitro assays, we demonstrated that DXO acts as an important suppressor of proliferation in BC. These findings suggest that DXO may function as a tumor suppressor in BC cell. Mechanically, cyclin D1 was confirmed as a downstream target gene and was negatively regulated by DXO. However, whether this was direct or indirect requires further investigation.

Based on the protein–protein analysis, we demonstrated that NPL4 interacted with DXO directly. As we expected, knockdown of NPL4 significantly upregulated DXO expression and the relationship between DXO and NPL4 explained how NPL4 regulated cyclin D1 mRNA expression. DXO was shown to be a critical downstream molecule of NPL4 and mediates NPL4 and cyclin D1 related cell proliferation. The NPL4/DXO/cyclin D1 axis may represent new mechanism of regulating BC cell growth.

## Conclusion

In conclusion, NPL4 is upregulated in BC, and promotes cell proliferation by binding directly to DXO and inducing its degradation, whereas DXO depresses cyclin D1 expression by decapping its mRNA (Fig. 7). NPL4 may have a therapeutic potential to suppress BC by inhibiting the DXO/cyclin D1 signaling pathway.

## Methods

### Tissue and cell lines

We collected 35 pairs of human primary BC tissue and the corresponding adjacent noncancerous bladder urothelial tissue the Second Hospital of Hebei Medical University from July 2015 to June 2018. All BC patients were histopathologically and clinically diagnosed and without chemotherapy or radiotherapy treatment. The study protocol was approved by the Ethics Committee of the Second Hospital of Hebei Medical University. Patients were obtained by written consent. The T24 BC cell line was purchased from ATCC (Rockville, Maryland). All Cells were cultured in Dulbecco’s modified eagle medium (Gibco, Beijing, China), which contained 10% fetal bovine serum (FBS) (Clark Bio, Claymont, DE, USA), 100 units/mL penicillin and 100 μg/mL streptomycin. Cells were incubated at 37 °C in a humidified incubator with 5% CO_2_.

### Cell transfection

All transfections were following Lipofectamine 2000 (Invitrogen) manufacturer’s instructions. si-NPL4 and si-DXO were purchased from GenePharma Co., Ltd (Shanghai, China). The overexpression vector of were purchased from GENEWIZ Company (Suzhou, China). Transfected cells were harvested and lysed for Western blotting analysis, and total RNA was extracted for quantitative reverse transcription polymerase chain reaction (qRT-PCR) analysis.

### RNA isolation and real-time PCR

Tissues and cultured cells were lysed using QIAzol Lysis Reagent (79306). Total RNA was extracted from the samples using a miRNeasy Mini Kit (217004; Qiagen) according to the manufacturer’s instructions. RNA quality was determined using a NanoDrop 2000. M-MLV First Strand Kit (Life Technologies) was used to cDNA synthesized with random hexamer primers. mRNAs were subjected to quantitative real-time polymerase chain reaction (qRT-PCR) using the Platinum SYBR Green qPCR Super Mix UDG Kit (Invitrogen) and the ABI 7500 FAST system (Life Technologies). Relative transcript expression levels were normalized to GAPDH and calculated using the 2^−ΔΔCt^ formula.

### MTT assay

Cell viability was detected by MTT [3-(4,5-dimethylthiazol-2-yl)-2,5-diphenyltetrazolium bromide] colorimetric assay. Briefly, T24 cells were plated in 96-well plates and treated with actinomycin (Act) for 0, 2, 4, and 8 h then 20 μL of MTT reagent (5 mg/mL; Sigma-Aldrich, St. Louis, MO, USA) was added into each well, incubated for 3–4 h, and the absorbance was measured at 495 nm using a microplate reader (Thermo Fisher, USA).

### Colony forming test

Colony forming test was used to detect cell viability. In brief, 100 cells/well T24 cells were seeded into 6-well plates and growed for 1 week. After fixing with a glacial acetic acid/methanol solution (1:3) and washing by phosphate-buffered saline (PBS), Colonies were stained with 0.5% crystal violet for 10 min then counted under a stereomicroscope using a 1-cm^2^ grid. Four squares from four quadrants were counted for each well.

### Western blot analysis

Cultured cells were lysed with lysis buffer. Equal amounts of protein were run on 10% SDS-PAGE and electrotransferred to polyvinylidene fluoride membranes (Millipore). After blocking with 5% milk in TTBS, membranes were incubated with primary antibodies. The following antibodies were used: anti-NPL4 (1:1000, ab224435), anti-cyclin D1 (1:1000, 60186-1-Ig), anti-DXO (1:1000, ab152135), and anti-β-actin (1:1000, sc-47778). Membranes were then incubated with the horse radish peroxidase-conjugated secondary antibody (1:5000, Rockland). The blots were detected by enhanced chemiluminescence Fuazon Fx (Vilber Lourmat), Fusion Capt Advance Fx5 software was used to captured the images.

### Coimmunoprecipitation (CoIP) assay

3 μg of antibodies and protein A-agarose were added to T24 cell lysates for 12 h at 4 °C. Then protein A-agarose–antigen–antibody complexes were collected by centrifugation. After washeding by IPH buffer, the extensive wash with lysis buffer, the immunoprecipitates were resolved by 10% SDS-PAGE, followed by Western blot analysis.

### Gene interaction networks

The gene interaction networks were identified using the GeneMANIA web server (http://www.genemania.org/) with default parameters.

### Statistical analysis

Data are presented as mean ± SEM. Differences between two group s analyzed by Student’s *t* test. Spearman’s correlation analysis was used to analyze the two genes correlation. Values of *P *< 0.05 were considered statistically significant.

## Data Availability

Not applicable.

## Abbreviations

BC: bladder cancer
NPL4: nuclear protein localization protein 4 homolog
VCP: valosin-containing protein
DXO: decapping and exoribonuclease protein
CoIP: coimmunoprecipitation
Act: actinomycin
qRT-PCR: quantitative real-time polymerase chain reaction
